# Fatal Dropsy after Scarlatina

**Published:** 1844-05-04

**Authors:** 


FATAL DROPSY AFTER SCARLATINA.

From M. Becquerel's work "On the Semeiology of the Urine," we learn that children dying with dropsy after scarlatina and without Bright's disease, present the following conditions on post-mortem examination: the subcutaneous and sometimes the deep inter-muscular cellular tissue is infiltrated with serosity; the peritoneum, and in some subjects the pleuræ and pericardium, contain serosity, generally straw-coloured and transparent: all the organs are remarkable for their pallor and anemia; some of them infiltrated with serosity—for example, in many cases the submucous cellular tissue of the coats of the intestine. The lung is almost always distinctly and considerably œdematous; the liver soft, flaccid, pale, and on incision giving issue to serum. The kidneys present peculiar appearances which are referrible to œdema of their substance, occurring in two degrees. In the first degree, the kidneys appear harder and more swollen than natural; the capsule may be completely separated; under it the tissue appears of a dull white colour (without any trace of a yellow hue) interrupted here and there with vascular streaks. The entire of the cortical substance, even that prolonged between the tubuli, appears of the same colour, and presents neither granular texture nor vascularity. The tubular substance has its natural form, and by its reddish hue stands on in strong relief from the surrounding pallid ground. Pressure forces out a certain quantity of serosity from the altered tissue: and when this is removed, the kidney loses its swollen and turgid aspect, and becomes soft and flaccid. This change may affect the entire or a part only of the cortical substance: in the latter case it would appear chiefly in the neighbourhood of the hylus. In the second and more advanced stage, the kidneys are decidedly larger and harder than natural; the capsule continues easily separable, the cortical substance is of a slightly yellowish white colour, or perfectly white and shining: no vascular appearance is visible, more serum can be forced out by pressure: the tubuli look paler, but still are obviously distinguished by colour from the surrounding cortical substance. Both kidneys are always affected: and it can only be regarded as a very advanced evidence of the general tendency to dropsy, like the infiltration of the liver, &c. The albumen in the urine in such cases appears to be furnished by the serosity pervading the renal tissue, which makes its way by a sort of infiltration or transudation into the excretory passages: be that as it may, M. Becquerel considers that his cases justify him in the inference that in the present kind of dropsy, the urine only contains albumen, when the kidneys participate in the general serous infiltration of the body. In some cases of dropsy after scarlatina, where the urine did not contain any albumen, he found the kidneys slightly paler than natural, but otherwise unaltered.—*Lon. Med. Times.*

